# The feasibility of MR elastography with transpelvic vibration for localization of focal prostate lesion

**DOI:** 10.1038/s41598-024-54341-0

**Published:** 2024-02-16

**Authors:** Hyo Jeong Lee, Soo Buem Cho, Jeong Kyong Lee, Jin Sil Kim, Chang Hoon Oh, Hyun Jin Kim, Hana Yoon, Hyun Kyu Ahn, Myong Kim, Yeok Gu Hwang, Hye Young Kwon, Moon Jung Hwang

**Affiliations:** 1 Department of Radiology, Ewha Womans University College of Medicine, Seoul, South Korea; 2 Department of Urology, Ewha Womans University College of Medicine, Seoul, South Korea; 3 Department of Orthopedic Surgery, Ewha Womans University College of Medicine, Seoul, South Korea; 4 Department of Radiology, Chungnam National University College of Medicine, Daejeon, South Korea; 5 GEHC, Seoul, Korea

**Keywords:** Diseases, Urology

## Abstract

We aimed to evaluate the feasibility of MR elastography (MRE) using a transpelvic approach. Thirty-one patients who underwent prostate MRE and had a pathological diagnosis were included in this study. MRE was obtained using a passive driver placed at the umbilicus and iliac crests. The shear stiffness, clinical data, and conventional imaging findings of prostate cancer and benign prostatic hyperplasia (BPH) were compared. Inter-reader agreements were evaluated using the intraclass coefficient class (ICC). Prostate MRE was successfully performed for all patients (100% technical success rate). Nineteen cancer and 10 BPH lesions were visualized on MRE. The mean shear stiffness of cancer was significantly higher than that of BPH (5.99 ± 1.46 kPa vs. 4.67 ± 1.54 kPa, p = 0.045). One cancer was detected on MRE but not on conventional sequences. Six tiny cancer lesions were not visualized on MRE. The mean size of cancers that were not detected on MRE was smaller than that of cancers that were visible on MRE (0.8 ± 0.3 cm vs. 2.3 ± 1.8 cm, p = 0.001). The inter-reader agreement for interpreting MRE was excellent (ICC = 0.95). Prostate MRE with transpelvic vibration is feasible without intracavitary actuators. Transpelvic prostate MRE is reliable for detecting focal lesions, including clinically significant prostate cancer and BPH.

## Introduction

Magnetic resonance elastography (MRE) is a phase contrast-based MRI technique that visualizes propagating mechanical waves and processes the data to develop quantitative images that represent intrinsic mechanical properties^1^. MRE is a non-invasive imaging modality that can be used to diagnose and manage diseases, such as cancer or fibrosis, with mechanical properties correlate well with tissue abnormalities. In MRE, a harmonically driven mechanical exciter generates shear waves, which are visualized and calculated to determine the shear modulus^1,2^.

The clinical value of MRE was first demonstrated in the assessment of hepatic fibrosis^3–5^. Thereafter, MRE has been widely studied for the detection of abnormalities in the breast^6^, brain^7^, lungs^8^, and heart^9^. There has also been considerable interest in the utilization of MRE for the assessment of the prostate. Current MRE techniques have limitations in delivering a sufficient level of shear wave amplitude to the prostate, which is located in the deep pelvis, in a patient-friendly manner. Several methods of generating shear wave vibrations in the prostate, including transurethral, transrectal, and transperineal approaches, have been investigated^10–12^. However, these methods can cause discomfort to patients and require additional devices or equipment.

Therefore, the aim of this study was to investigate the feasibility of prostate MRE using transpelvic vibration without intracavitary actuators.

## Material and methods

### Study population

**Figure 1:**
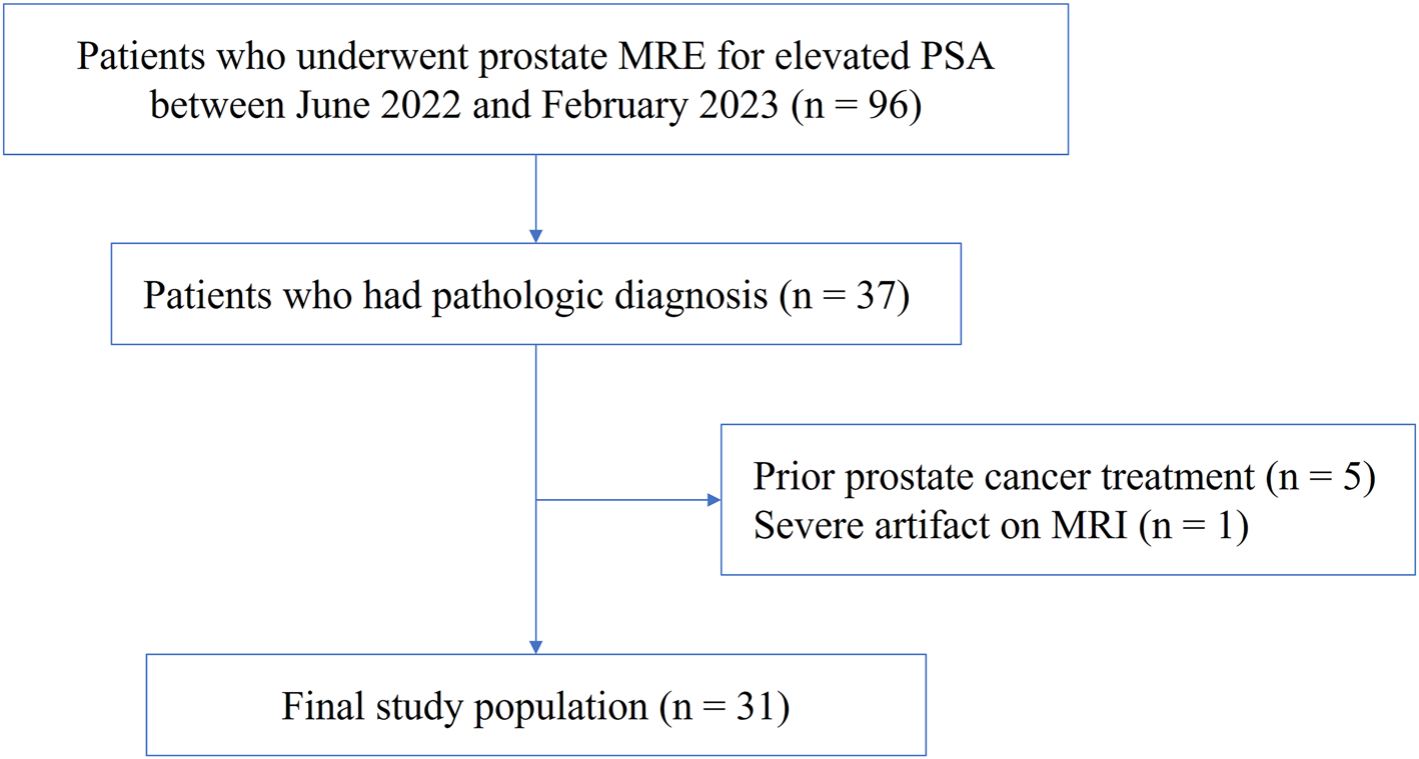
Flowchart of patient inclusion.

This retrospective single-center study was approved by the institutional review board of Ewha Womans University (IRB No. 2023-07-043-002), which waived the need for obtaining informed consent from the patients. All methods were performed in accordance with the relevant guidelines and regulations. In June 2022, we added MRE to the conventional multiparametric prostate MRI protocol utilized at our institution. A total of 96 consecutive patients underwent MRE in our institution between June 2022 and February 2023. Of these 96 patients, we included 37 whose prostatic pathological findings were confirmed through prostate biopsy or surgery. Five patients were excluded because they had previously received treatments, such as androgen deprivation therapy (ADT, n = 2), holmium laser enucleation of the prostate (HoLEP, n = 2), or transurethral resection of the prostate (TURP, n = 1). One patient was excluded due to the detection of severe metal artifacts on MRI caused by a hip prosthesis. Finally, 31 patients (mean age, 69.1 ± 7.9 years; mean body weight, 69.6 ± 10.0 kg) were included (Fig. 1). We recorded the patients’ age, body weight, serum prostate-specific antigen (PSA) levels, and pathological findings.

### Imaging technique

MRI was performed using a 3.0-T system (SIGNA Architect; GE Healthcare) with spine posterior 40ch AIR and 16ch cardiac coils. The conventional prostate MRI parameters for the axial, coronal, and sagittal T2-weighted fast spin-echo sequences were as follows: field of view (FOV), 200 mm; repetition time (TR)/echo time (TE), 2722–2920 ms/102.0–110.0 ms; flip angle (FA), 111°–152°; slice thickness, 3.0 mm. The parameters for the axial T1-weighted fast spin-echo sequence were as follows: FOV, 200 mm; TR/TE, 620 ms/8 ms; FA, 111°; slice thickness, 3.0 mm. The parameters for the transverse diffusion-weighted imaging (DWI) sequence of single shot echo planar imaging were as follows: FOV, 200 mm, TR/TE 5000 ms/70.0–80.0 ms; slice thickness, 4.0 mm; b values 0, 1000, and 1400 s/mm^2^ The ADC map was automatically calculated using a monoexponential curve fit of only 2 b values (high b values of 1400 s/mm^2^, low b value of 0 s/mm^2^). The parameters for the transverse 3D dynamic contrast-enhanced sequence with a temporal resolution of 11 s after intravenous injection of 0.1 mmol/kg gadolinium chelate at a rate of 1.5 mL/s were as follows: FOV, 240 mm; TR/TE, 5.12 ms/2 ms; FA, 12°; slice thickness, 1.5 mm.

### MR elastography

MRE was performed at the end of the examination of standard MRI protocol. The MRE hardware consists of two parts: active and passive drivers. An active driver generates continuous acoustic vibrations at 60 Hz or 90 Hz, which synchronize the imaging spin-echo EPI pulse sequence. The acoustic wave is transmitted to the passive driver, the drum, via a flexible vinyl tube. For the MRE procedure in the present study, the passive driver was placed against the umbilicus and the iliac crests, which were expected to propagate shear waves to the prostate. The hardware was designed and manufactured by Resoundant, a Mayo Clinic-affiliated company.

A free-breathing spin echo EPI-based phase contrast sequence was used to image the different phase displacements correlated with the stiffness per tissue. The pulse sequence is modified by an oscillating motion-encoding gradient (MEG). The active driver is triggered by the pulse such that the MEG is synchronized with the external acoustic wave. The duration of the MEG is the same as that of the mechanical vibration. A phase shift occurs in the MR signal, which is correlated with the mechanical excursion. These phase changes were inverted to the stiffness voxel by voxel using multi-model direct inversion algorithms (MRMS, 2017, Preliminary Comparison of Multi-scale and Multi-model Direct Inversion Algorithms for 3.0-T MR Elastography). A higher driver frequency value results in a shorter wavelength and better resolution but at the expense of less wave penetration. In the present study, we used 90 Hz instead of 60 Hz, which is more suitable for liver imaging, because the prostate is a smaller organ than the liver.

The MRE acquisition parameters used were as follows: TR/TE, 2000/70.5; FOV, 240 mm; matrix, 80 × 80; slice thickness, 3.0 mm (acquisition voxel size, 3.0 × 3.0 × 3.0; reconstructed voxel 1.5 × 1.5 × 3.0); chemical fat saturation; NEX, 8; bandwidth, 250 kHz; temporal phase, 4; MEG frequency, 90 Hz; driver frequency, 90 Hz; driver amplitude, 80%; MEG direction, Z (slice); driver cycle per trigger, 12; MENC, 18 um/rad; parallel imaging; ASSET; acceleration factor, 2 with a free-breathing scan time of 2 min 16 s.

### Imaging analysis

One radiologist with seven years of experience in imaging interpretation (H.J.L.) who was blinded to the clinical results interpreted the multiparametric prostate MRI findings. The volume of the prostate gland was calculated using axial and sagittal T2-weighted images (T2WI). Following the PI-RADS committee guidelines, the maximum longitudinal diameter (A) and maximum anteroposterior diameter (B) were measured on mid-sagittal T2WI, and the maximum transverse diameter (C) was measured on axial T2WI. The volume was then calculated using the formula: volume = A × B × C × 0.523. When a focal lesion was observed, the location, size, and Prostate Imaging Reporting and Data System (PI-RADS) score of the lesion were evaluated according to the guidelines of the PI-RADS version 2.1. Another reader (H.J.K.) who was blinded to the results scored PI-RADS, and these data were utilized to evaluate inter-observer agreement.

For the MRE analysis, the mean shear stiffness of each tumor was calculated using a manually drawn region of interest (ROI). The ROI was placed by a reader (with 15 years of experience in imaging interpretation) who was blinded to the final diagnosis (S.B.C.). We conducted the ROI assessment in the magnitude image by measuring a single slice in which the lesion appeared largest. The ROI was drawn in a circular shape, and its size was approximately 100 mm^2^. The ROIs were copied to the stiffness map, allowing for the calculation of stiffness values measured in kilopascals. Another reader (H.J.L.) measured mean shear stiffness in the same manner and utilized the data for inter-observer agreement analysis.

### Statistical analysis

The Mann–Whitney U test and Fisher’s exact test were used for the comparison of continuous and categorical variables, respectively, between groups. Inter-observer agreement was evaluated using the Cohen kappa coefficient for PI-RADS and intraclass coefficient class (ICC) for mean shear stiffness. ICC and κ values < 0.4, 0.4–0.6, 0.6–0.8, and > 0.8 were considered to indicate poor, moderate, good, and excellent agreement, respectively. Statistical significance was set at p < 0.05. SPSS version 25.0 (IBM Corp.) and MedCalc Statistical Software version 22.009 (MedCalc Software Ltd.) were used for all statistical analyses.

## Results

**Figure 2:**
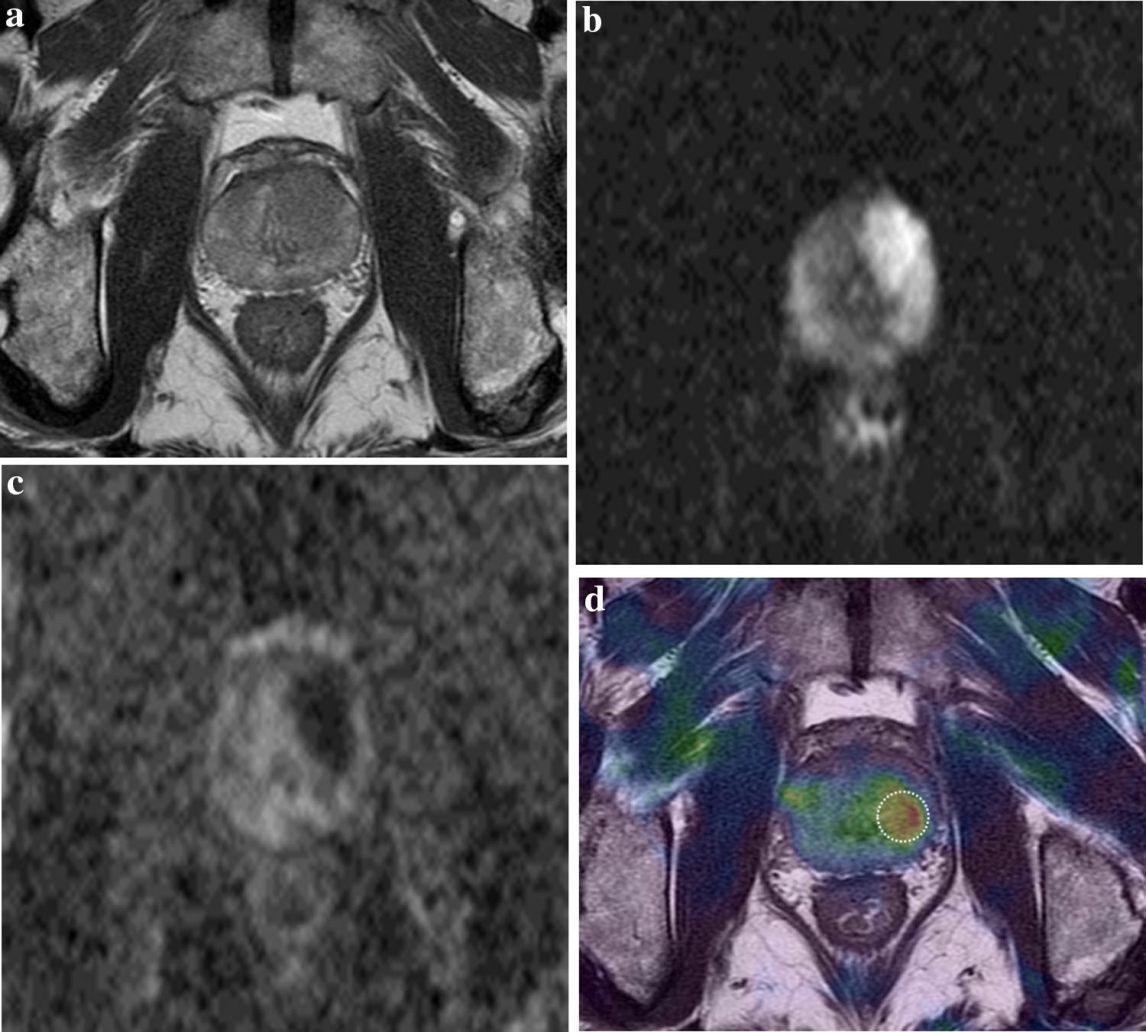
A 69-year-old man with prostate cancer confirmed after radical prostatectomy. (**a**) T2-weighted axial MR image showing a low-signal intensity lesion in the left apical transitional zone of the prostate. (**b, c**) Diffusion-weighted image (b = 1500 s/mm^2^) and ADC map show diffusion restriction in the corresponding area (ADC value, 0.528 × 10^−3^ m^2^/s). (**d**) Elastogram shows that the mean stiffness of the tumor is 6.218 kPa.

**Figure 3:**
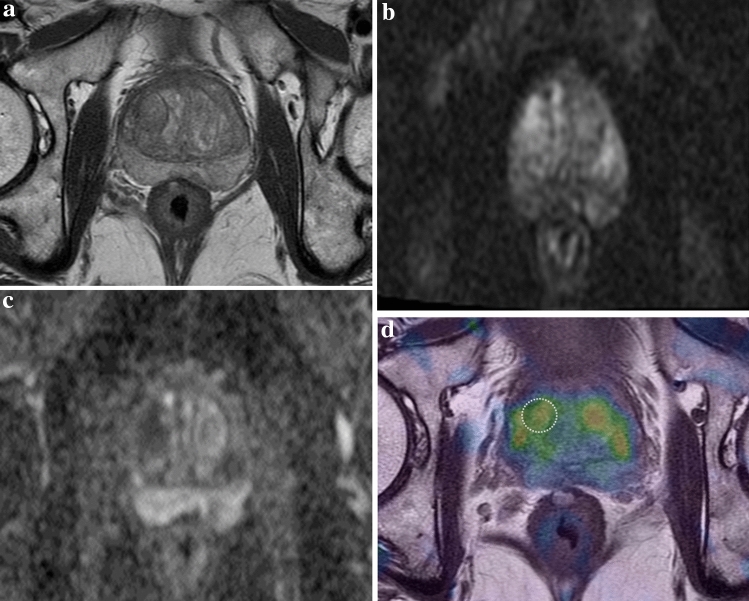
A 75-year-old man with benign prostatic hyperplasia confirmed using MRI-US fusion targeted biopsy. (**a**) T2-weighted axial MR image showing a well-encapsulated hypointense nodule in the right mid-transitional zone of the prostate. (**b**, **c**) Diffusion-weighted image (b = 1500 s/mm^2^) and ADC map show diffusion restriction in the corresponding area (ADC value, 0.735 × 10^−3^m^2^/s). (**d**) Elastogram shows that the mean stiffness of the tumor is 3.982 kPa.

**Figure 4:**
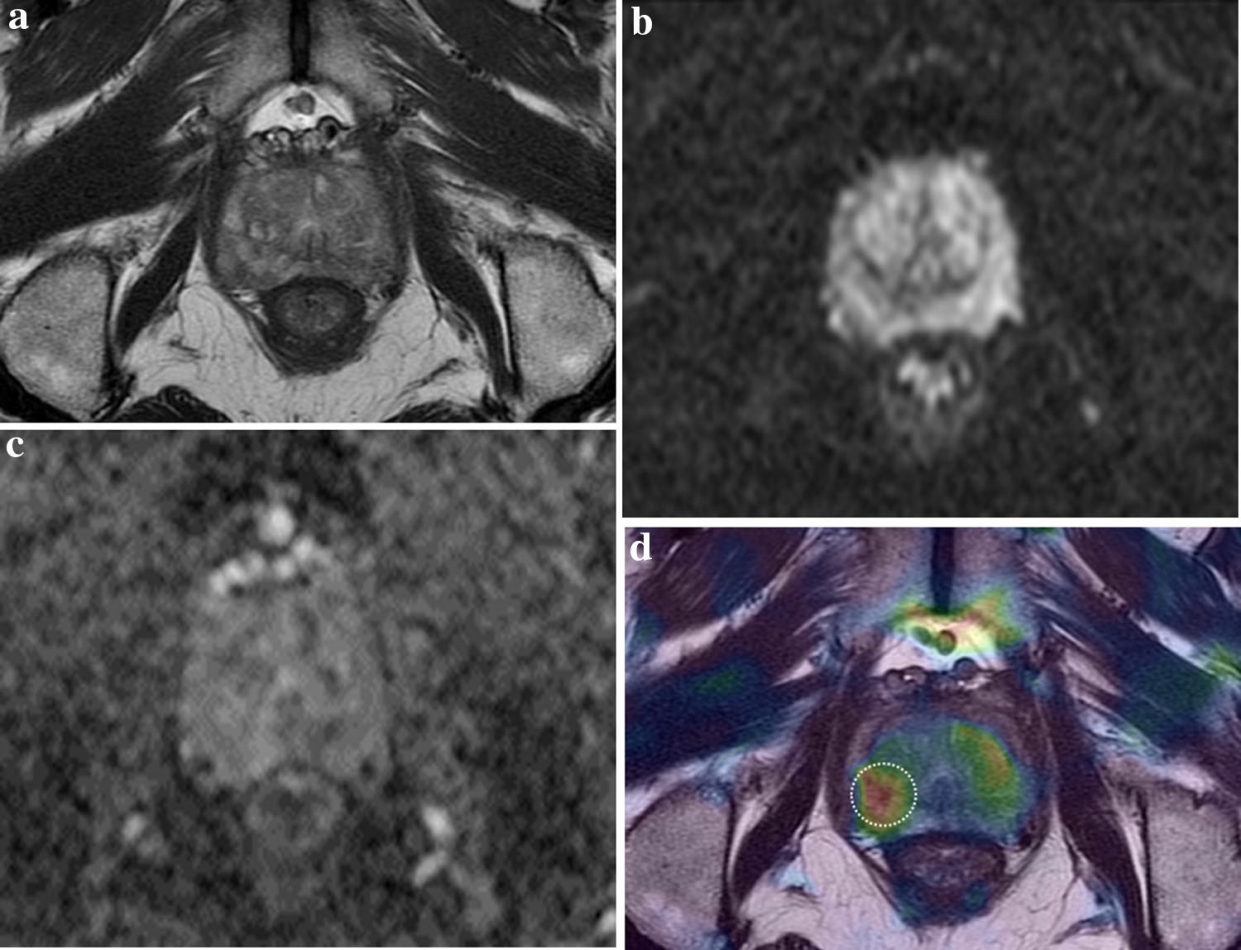
A 64-year-old man was diagnosed with prostate cancer in the right lobe after radical prostatectomy. (**a**–**c**) There is no evidence of clinically significant prostate cancer on conventional prostate MRI (from left to right: axial T2WI, DWI, and ADC). (**d**) Elastogram shows a tumor in the right apical peripheral zone of the prostate, with a mean stiffness of 7.512 kPa.

MRE of the prostate was successfully performed for 31 patients (100% technical success rate). The patients tolerated the examination well and no adverse effects were reported. Twenty-nine focal lesions were visualized on the wave images of 22 patients. The size of the ROI ranged from 92.5 to 105.2 mm^2^, with an average of 99.6 ± 4.1 mm^2^. Nineteen lesions were confirmed as prostate cancer, whereas 10 were diagnosed as benign prostatic hyperplasia (BPH). The mean shear stiffness of prostate cancer lesions was significantly higher than that of BPH tissues (5.99 ± 1.46 kPa vs. 4.67 ± 1.54 kPa, p = 0.045) (Table 1). The PI-RADS score of prostate cancer was significantly higher than that of BPH lesions (p < 0.001). In addition, most of the cancer lesions (94.7%) had a PI-RADS score ≥ 3. The mean PSA level in prostate cancer was higher than that in BPH (19.7 ± 28.3 ng/mL vs. 7.4 ± 3.9 ng/mL, p = 0.062). Representative examples of prostate cancer and BPH are shown in Figs. 2 and 3, respectively. One cancerous lesion was detected on MRE but not conventional MRI sequences (Fig. 4).

**Table 1:** Comparison of the visualization of prostate cancer and BPH which were visualized on MRE.

	Prostate cancer (n = 19)	BPH (n = 10)	p value*
Shear stiffness (kPa)	5.99 ± 1.46	4.67 ± 1.54	0.045
PI-RADS ≥ 3	18 (94.7)	0 (0.0)	< 0.001
PSA (ng/mL)	19.7 ± 28.3	7.4 ± 3.9	0.062

*BPH* Benign prostatic hyperplasia, *MRE* Magnetic resonance elastography, *PI-RADS* Prostate Imaging Reporting and Data System, *PSA* Prostate-specific antigen.

Categorical variables are expressed as numbers of patients, with percentages in parentheses. Continuous variables are expressed as mean ± standard deviation.

*Continuous data were assessed using the Mann–Whitney U test, whereas categorical data were assessed using Fisher’s exact test.

Six pathologically confirmed prostate cancer lesions were not visualized on MRE, and one of them was not interpreted as a suspected cancer lesion on conventional MRI. The mean size of the cancers that were not visible on MRE was significantly smaller than that of cancers that were visible on MRE (0.8 ± 0.3 cm vs. 2.3 ± 1.8 cm, p = 0.001) (Table 2). In addition, the PSA level in cancers that were visible on MRE was higher than that in cancers that were not visible on MRE (19.7 ± 28.3 ng/mL vs. 5.6 ± 1.6 cm, p = 0.030). PI-RADS and Gleason score tends to be higher in MRE-visible cancers than in MRE-nonvisible cancers, but there was no statistical significance (PI-RADS; 4.4 ± 0.8 vs. 3.7 ± 0.8, p = 0.066, Gleason score; 7.5 ± 0.8 vs. 6.8 ± 0.8, p = 0.110). Also, there was no statistically significant difference in total prostate volume between cancers that were visible on MRE and those that were not visible on MRE (p = 0.121).

**Table 2:** Comparison of cancers that were visible on MRE with those that were not.

	MRE-visible cancer (n = 19)	MRE-nonvisible cancer (n = 6)	p value*
Lesion size (cm)	2.3 ± 1.8	0.8 ± 0.3	0.001
PSA (ng/mL)	19.7 ± 28.3	5.6 ± 1.6	0.030
PI-RADS	4.4 ± 0.8	3.7 ± 0.8	0.066
Gleason score	7.5 ± 0.8	6.8 ± 0.8	0.110
Prostate volume (mL)	50.5 ± 31.2	33.2 ± 13.0	0.121

*MRE* Magnetic resonance elastography, *PSA* Prostate-specific antigen.

*Data were assessed using the Mann–Whitney U test.

Analysis of preliminary diagnostic performance revealed that MRE has a sensitivity of 76.0%, specificity of 37.5%, positive predictive value of 65.5%, negative predictive value of 50.0%, and accuracy of 61.0% for the prediction of prostate cancer.

Interobserver agreement was good for PI-RADS (κ = 0.740) and excellent in elastogram analysis (ICC = 0.95, 95% CI 0.90–0.98).

## Discussion

This preliminary study was conducted to investigate the feasibility of prostate MRE with transpelvic vibration for the assessment of the focal prostate lesions. MRE images were successfully obtained for all patients. Nineteen of 25 (76.0%) prostate cancer lesions were visualized on MRE. One lesion was only depicted on MRE, but not on T2WI or DWI. The mean shear stiffness of prostate cancer lesions was significantly higher than that of BPH lesions (5.99 ± 1.46 kPa vs. 4.67 ± 1.54 kPa, p = 0.045). The results of ex vivo studies have indicated that the stiffness of prostate cancer tissues is approximately two to three-times greater than that of normal tissues^13–15^. A previous study using MRE depicted that prostate cancer is harder than BPH, which correlates with the results of our study^16^. A key mechanism of tumor stiffening is the excessive deposition of extracellular matrix components, particularly type I collagen^17^.

As the mechanical properties of prostate tissues can be used as diagnostic parameters, elastography has been investigated for the localization of prostate cancer. Most previous studies were focused on ultrasound elastography because of its widespread accessibility and ability to provide real-time imaging^18,19^. However, MRE offers an advantage over ultrasound elastography because it can acquire a complete three-dimensional wave field. This comprehensive data acquisition allows for complete inversion of the wave equation in MRE, potentially enhancing its accuracy^20^. In addition, the reproducibility and reliability of MRE are superior to those of ultrasound elastography because the MR technique is operator-independent. However, the limitation of MRE is that the shear waves are significantly attenuated as they propagate through soft tissue. Arani et al. demonstrated the feasibility of utilizing intracavitary actuators for performing MRE through the urethra and rectum^10,11^. The advantage of intracavitary actuators is that they require shorter penetration depths of shear waves than external approaches. They also allow higher-frequency shear waves to reach the prostate and increase spatial resolution. Although the outcomes of transurethral or transrectal MRE are promising, the invasive nature of the procedure hinders its routine diagnostic application. Several studies have attempted to apply the transpelvic approach for prostate MRE, and they have successfully implemented it^16,21^. However, they utilized an advanced MRE protocol specifically developed for their investigations. In our study, we opted for a conventional product protocol for MRE, ensuring practical applicability in our daily practice.

Moreover, in terms of BPH, ultrasound elastography has been used to analyze the severity of symptoms and prostatic elasticity in a previous study^22^. However, no study has been conducted to investigate the correlation between BPH and stiffness using MRE to date. The findings of the present study indicate that MRE using transpelvic vibration could play a valuable role in the detection of prostate cancer and the evaluation of BPH.

In the present study, 6 of 25 cancer lesions were not detected on MRE. The size of all cancers that were not visible on MRE was less than 1 cm, with a mean size of 0.8 cm. These lesions were significantly smaller than the cancers that were visible on MRE. Owing to the lower signal-to-noise ratio in MRE than in conventional MRI, the detection of small lesions on MRE is inevitably limited. However, in the present study, the tumors that were not visible on MRE were small and had significantly lower PSA levels than those that were visible. This indicates that MRE has sufficient diagnostic value for detecting clinically significant prostate cancer. Remarkably, one tumor was visualized on MRE but not on conventional MRI.

Inter-observer agreement between the two radiologists in the interpretation of MRE findings was excellent (ICC = 0.95). According to a previous systematic review, the inter-observer agreement for the PI-RADS version 2.1 ranges from fair to good, with a κ (kappa) value of 0.310–0.673^23^. It is well known that the greatest limitation of the PI-RADS is its high inter-reader variability. Given that MRE is an objective modality that provides stiffness measurements for quantitative analysis, it can reduce variability and increase the accuracy of MRI reporting.

This study has several limitations. First, the pathological diagnoses of most patients (23/31) was based on prostate biopsy findings rather than on radical prostatectomy findings; therefore, the actual prostate pathology in each case may not have been accurately evaluated. However, more than two-thirds of the biopsies were performed using the MRI-US fusion platform (Artemis), which has been reported to be more accurate than a systematic biopsy^24^. Second, due to the retrospective nature of the study, we could not evaluate the reproducibility of the MRE protocol. In addition, we did not include healthy volunteers as study subjects; therefore, we were unable to determine the absolute value of elasticity in prostate cancer tissues. Thus, a prospective study with healthy volunteers is needed to investigate the reproducibility of MRE and increase the quantitative accuracy of lesion elasticity.

In conclusion, the results of this study demonstrated the feasibility of prostate MRE using a transpelvic approach. This study provides basic data for future studies on prostatic elasticity in patients with prostate cancer and BPH.

## Data Availability

The datasets used and/or analyzed during the current study are available from the corresponding author upon reasonable request.
